# BPGA- an ultra-fast pan-genome analysis pipeline

**DOI:** 10.1038/srep24373

**Published:** 2016-04-13

**Authors:** Narendrakumar M. Chaudhari, Vinod Kumar Gupta, Chitra Dutta

**Affiliations:** 1 Structural Biology & Bioinformatics Division, CSIR- Indian Institute of Chemical Biology, 4, Raja S. C. Mullick Road, Kolkata 700032, India

## Abstract

Recent advances in ultra-high-throughput sequencing technology and metagenomics have led to a paradigm shift in microbial genomics from few genome comparisons to large-scale pan-genome studies at different scales of phylogenetic resolution. Pan-genome studies provide a framework for estimating the genomic diversity of the dataset, determining core (conserved), accessory (dispensable) and unique (strain-specific) gene pool of a species, tracing horizontal gene-flux across strains and providing insight into species evolution. The existing pan genome software tools suffer from various limitations like limited datasets, difficult installation/requirements, inadequate functional features etc. Here we present an ultra-fast computational pipeline BPGA (Bacterial Pan Genome Analysis tool) with seven functional modules. In addition to the routine pan genome analyses, BPGA introduces a number of novel features for downstream analyses like core/pan/MLST (Multi Locus Sequence Typing) phylogeny, exclusive presence/absence of genes in specific strains, subset analysis, atypical G + C content analysis and KEGG & COG mapping of core, accessory and unique genes. Other notable features include minimum running prerequisites, freedom to select the gene clustering method, ultra-fast execution, user friendly command line interface and high-quality graphics outputs. The performance of BPGA has been evaluated using a dataset of complete genome sequences of 28 *Streptococcus pyogenes* strains.

With the advent of high-throughput low-cost sequencing technologies and metagenomic techniques, the field of microbial genomics has experienced a paradigm shift from single or few genome analyses to large-scale comparisons of hundreds to thousands of genomes. One of the important outcomes of such multi-genome studies is the concept of the pan-genome - a term coined by Tettelin *et al.* in 2005 to refer to the complete inventory of genes in a particular species [in Greek, pan (παν) means ‘whole’]^1^. A study of seven *Streptococcus agalactiae* genomes by Tettelin *et al.* demonstrated that strains of a bacterial species might differ substantially in their gene content and total gene pool of a species might be orders of magnitudes larger than the gene content of any single strain^1^. It is, therefore, rational to describe a bacterial species by its pan-genome, which includes a core genome containing genes shared by all strains, a dispensable genome containing accessory genes that exist in two or more strains and genes unique to single strains (also known singletons). The core genes are responsible for the basic aspects of the biology of the species and its major phenotypic traits; while the accessory genes and singletons usually pertain to supplementary biochemical pathways and functions that may confer selective advantages such as ecological adaptation, virulence mechanisms, antibiotic resistance, or colonization of a new host. The pan genome analyses represent a new approach to species definition and provide a framework for estimating and/or modeling the genetic diversity of the study group.

During last ten years, pan genomic studies have been conducted on nearly fifty bacterial species, which include model organisms like *Escherichia coli* and members of normal human flora like *Lactobacillus paracasei* as well as a number of pathogens like *Haemophilus influenza*, *Coxiella burnetii*, *Streptococcus pneumonia*, *Yersinia pestis* etc.^2^,^3^,^4^,^5^,^6^,^7^. Traditionally defined at the species level, the pan genomic approach has later been implemented also at higher levels of phylogenetic resolution ranging from genus to phylum and beyond. Genus level studies were carried out on *Streptococcus*, *Salmonella*, *Prochlorococcus* etc., and the pan genome study of the *Chlamydiae* is an example of phylum level studies. Recently, Lapierre & Gogarten has extended the concept of the pan genome to the entire bacterial domain^8^,^9^,^10^,^11^,^12^. The concept has also been implemented in viral, plant and fungal genome studies^13^,^14^,^15^,^16^,^17^. Pan genome analyses have provided valuable insight into genome dynamics, population structure, species evolution, niche specialization, pathogenesis, drug resistance and many other features of the microbial world^1^,^18^,^19^,^20^,^21^,^22^. It has also been exploited for development of vaccines against bacteria^23^.

A number of free tools and web servers are available for pan genome analysis, but each of them suffers from one or the other limitations, leaving rooms for further improvement. For instance, PanOCT, being a web-based database, is applicable only to a limited number of species. Panseq, PanGP and Roary provide few functional features and hence, lack in downstream analyses. PGAP and ITEP include a number of functional modules, but are much slower in speed^24^,^25^,^26^,^27^,^28^,^29^. There has been, therefore, a pressing need for development a new computational pipeline, which will not only offer fast and efficient formalisms for construction of the pan genome through clustering of orthologous gene families and but also enable various downstream analyses such as mapping of the core, accessory & unique genes to various COG categories and/or KEGG pathways, phylogenetic analysis, *in silico* multi locus sequence typing (MLST) and other relevant analyses. The pipeline should also provide options for selecting from different tools available for orthologous clustering and formation of binary gene presence/absence matrix. An option for applying the tools to a subset of the total dataset may facilitate identification of exclusive genetic features that can discriminate between different serological, ecological or pathogenic groups.

In this context; we have developed an ultra-fast computational pipeline BPGA (Bacterial Pan Genome Analysis Tool) with seven functional modules for comprehensive pan genome studies and downstream analyses. In addition to all types of analyses offered by currently available tools, this pipeline contains certain novel features like Exclusive Gene Family Analysis, KEGG Pathway Analysis, GC Content Analysis, Subset Analysis etc. Other notable features of BPGA includes minimum running prerequisites, ease of handling, user friendly command line interface, freedom for user to select method for clustering, high quality image output and efficiency in terms of time cost.

## Methods

BPGA is written in perl programing language but complied in executable files for both Windows and Linux so that no module installation is required. The perl code is freely available to make BPGA system independent. Dependencies like MUSCLE and rsvg-convert are also provided inside the installer, required for sequence alignments and tree generation^30^. Another prerequisite *gnuplot 4.6.6* which is required for plotting results into graphs; should be manually installed on the system.

BPGA performs pre-processing step to prepare sequence data for clustering. BPGA then runs USEARCH for fastest clustering (using 50% sequence identity cut-off by default; user may change this cut-off value). The clustered output is processed to generate tab delimited gene presence absence binary matrix (pan-matrix) which is then used for pan-genome profile calculations with iterations (default 20 or user defined) as well as pan genome based phylogeny. MUSCLE is used to align concatenated core genes to generate phylogeny tree based on core genome and MLST based on user selected housekeeping genes. For assigning COG and KEGG IDs, best hits with respective reference databases obtained from ublast function of USEARCH are used. Gnuplot is used for plotting all the results as high quality pdf images. Details about each step are provided in Table 1.

### BPGA Algorithm

BPGA performs pan genome analysis in stepwise manner using its core modules. The steps include:

#### Input preparation for clustering

Three types of input files can be processed by BPGA:

1. *GenBank file*: BPGA processes GenBank (.gbk) files for orthologous cluster analysis and generates an *input file* that may be used as an input for USEARCH, CD-HIT or OrthoMCL for clustering proteins.
2. *Protein Sequence File*: BPGA is also able to process protein sequence files (Standard NCBI/HMP fasta formats, .faa/.fsa or any other fasta format) for orthologous cluster analysis and generates an *input file* that may be used as an input for USEARCH, CD-HIT and OrthoMCL.
3. *Binary (1/0) Matrix*: BPGA processes tab-delimited binary (1/0) matrix file generated by any other tool, for pan-genome profile analysis.

#### Orthologous clustering of functional genes

Input files created by *Input Preparation* step are used to generate orthologous gene/protein clusters, which are then processed for downstream pan-genome analyses. For clustering gene families, user may select from any of the three clustering tools- USEARCH, CD-HIT and OrthoMCL. All three clustering tools generate almost similar results, but USEARCH, being the fastest among the three tools, has been integrated as the default clustering tool in BPGA. By default, 50% sequence identity is taken as the cut-off value for orthologous clustering, but users can set this cut-off value.

#### Functional modules

BPGA has seven functional modules. These include,

##### (i) Pan-genome profile analysis

**Figure 1:**
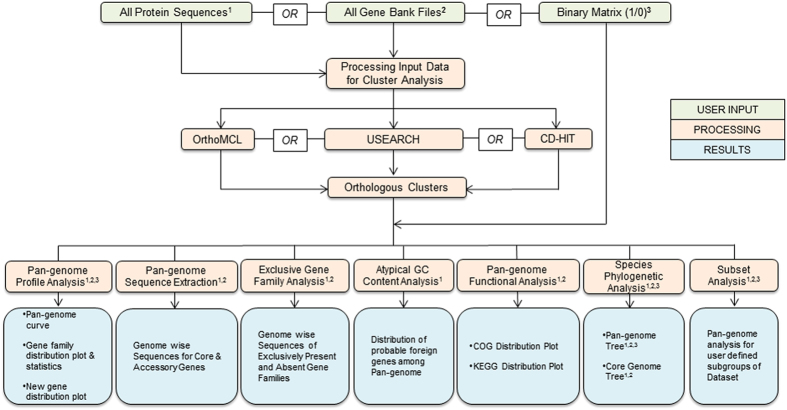
BPGA workflow.

This yields three types of graphical outputs – pan and core genome curves, frequency distribution of different gene families within the genomes under study and number of new genes added to the pan genome by each genome. The pan genome curve is generated by plotting the total number of distinct gene families against the number of genomes considered (Fig. 1c). Similarly, the number of shared gene families is plotted against the number of genomes in order to generate the core-genome plot that depicts the trend in contraction in the core genome size with sequential addition of more genomes (Fig. 1c). BPGA calculates the pan-genome size and core genome size for given *n* genomes. In order to avoid any bias in the sequential addition of new genomes, random permutations are carried out in the order of addition of genomes and median values of the total number of distinct gene families and number of shared gene families are taken respectively as the size of pan-genomes and core genomes. Number of permutation may be defined by the user. There is a provision for 500 permutations at each step of genome addition. But by default, 20 random permutations of genomes are carried out, as it has been observed that higher number of permutations does not alter the median values of pan genome and core genome sizes significantly.

The pan-genome size and core genome size after addition of each genome is calculated using following formulas,


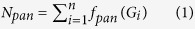



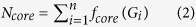


Where, G_*i*_ represents *i*^th^ gene family, *n* is the total number of distinct gene families obtained from the entire dataset and pan/core genome size, (*N*_*pan*_*/N*_*core*_) represents the size of the pan/core genome after addition of *n*^th^ genome from the dataset.

*f*_pan_ (G_*i*_) = 1, if one or more genomes among the *n* genomes contain at least one member of the gene family G*i*

*f*_pan_ (G_*i*_) = 0, if none of the *n* genomes contain a member of the gene family G*i*

*f*_core_ (G_*i*_) = 1, if all of the *n* genomes contain at least one member of G*i*

*f*_core_ (G_*i*_) = 0, if at least one of the *n* genomes does not contain any member of G*i*.

The power-law regression model for the pan-genome data and an exponential curve fit model in case of the core genome data.


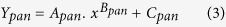



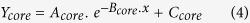


Where, *A*_*pan*_, *B*_pan_, *C*_*pan*_ *and A*_*core*_, *B*_*core*_, C_core_ are the fitting parameters. *Y*_*pan*_ and *Y*_*core*_ are calculated pan-genome and core genome sizes respectively.

##### (ii) Pan-genome sequence extraction

This module identifies core, accessory and unique protein families and extracts the representative (seed) protein sequence from each orthologous family. For a dataset of *n* genomes, the gene families with members from all of the *n* genomes are core genes, gene families having members from *x* genomes, where 1 < *x *< *n*, are accessory genes and those having members in any one particular genome of the dataset are unique genes or singletons.

##### (iii) Exclusive gene family analysis

This identifies the orthologous protein families that contain genes exclusively from a specific genome of the dataset (i.e., unique genes or singletons). It can also identify orthologous families that contain genes from all genomes except one specific genome (exclusively absent genes/proteins). The module also extracts the protein sequences of such exclusively present/absent families.

##### (iv) Atypical GC content analysis

This is an unique feature of BPGA, which extracts sequences of the genes, the G + C-contents of which deviate from the average G + C-content of the respective genomes by more than two or three times of the standard deviation, as follows,


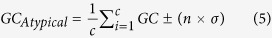


Where *GC*_*Atypical*_is the Atypical GC cut-off, c is the number of coding sequences in each genome, GC is G + C Content of *i*^th^ gene and σ is standard deviation in GC content within the genome and *n* = 2 or 3 (as defined by the user, 2 being the default value).

These genes are then categorized as the core, accessory and unique genes.

##### (v) Pan-genome functional analysis

This module explores the COG and KEGG pathway distribution. The COG and KEGG ids are assigned to all representative protein sequences from each orthologous protein cluster based on the protein BLAST against reference COG and KEGG databases. Then, the percentage frequencies of COG and KEGG categories are calculated for core genes, accessory genes and singletons (strain specific genes) and using *gnuplot*, outputs are generated in the form of histograms.

##### (vi) Species phylogenetic analysis

BPGA performs the evolutionary analysis based on concatenated core gene alignments and binary (presence/absence) pan-matrix. Gene matrix is calculated using similarity or dissimilarity in contribution of genes to orthologous gene clusters. For core genome based phylogenetic tree, BPGA first extracts the protein sequences (excluding paralogs) from 20 random orthologous gene clusters to generate core genome phylogeny tree and also performs *in silico* MLST analysis on user selected housekeeping genes. BPGA automates multiple sequences alignments using MUSCLE. All alignments were concatenated and a neighbor-joining phylogenetic tree was constructed.

##### (vii) Subset analysis

BPGA has the option for selecting a smaller subset of the original dataset for analysis, i.e., sub-grouping of genomes on the basis of their habitats, life-styles, morphology or virulence/symbiotic phenotypes etc. Such analyses may enable identification of genes exclusively present or absent in a specific sub-group along with routine pan genome analysis for all the sub-groups.

## Results

### Software features

BPGA is a computational pipeline, designed to automate the complete pan-genome study and downstream analyses of prokaryotic sequences. As shown in the work flow diagram (Fig. 1), BPGA has seven functional modules: (i) *Pan Genome Profile Analysis*, (ii) *Pan Genome Sequence Extraction*, (iii) *Exclusive Gene Family Analysis*, (iv) *Species Phylogenetic Analysis*, (v) *Pan Genome Functional Analysis*, (vi) *Atypical GC Content Analysis* and (vii) *Subset Analysis*.

BPGA performs pre-processing step to prepare sequence data for clustering. It’s the only pan genome pipeline that allows the user to select from three different tools for protein/gene clustering–USEARCH, CD-HIT or OrthoMCL, the first one being the default clustering tool due to its ultra-fast clustering capability^31^,^32^,^33^. The clustered output is processed to generate binary gene presence/absence matrix (pan-matrix) that is used for further downstream analyses by the functional modules of the pipeline with user defined parameters.

First three modules are integrated as a single step called *Default Pan Genome Analysis*, which includes classification of orthologous clusters into core, accessory and unique genes, extraction of their sequences, construction and extrapolation of the core and pan genome plots and identification of gene families that are exclusively present or absent (i.e., gene gain/loss) in a specific genome or subset of genomes.

The module on *Species Phylogenetic Analysis* provides options for construction of trees on the basis of pan matrix, core genome alignment and *in silico* Multi Locus Sequence Typing (MLST) based on user selected housekeeping genes^34^. The module for *Pan Genome Functional Analysis* performs the COG and KEGG assignments for representative sequences of all orthologous gene families and performs comparative functional analysis for core, accessory and unique set of genes. The module of *Atypical GC Content Analysis* – a unique feature of BPGA, extracts the sequences of genes having atypical G + C-contents i.e. genes with considerably higher or lower G + C-contents from the average G + C-content of the respective genomes (i.e., potential cases of horizontal transfer)^35^,^36^. These potential alien sequences are then categorized as the core, accessory or unique genes. Another unique feature of BPGA is the provision for analyzing different subsets of data–the *Subset Analysis* module- where the entire analysis can be carried out on a user-defined subset of the genomes, which may be selected on the basis of any specific feature of the respective organisms such as their morphology, pathogenic phenotypes or niche specialization^37^. Comparisons of BPGA features with other available tools are enlisted in Table 2.

### Evaluation of BPGA features with *Streptococcus pyogenes* test dataset

To evaluate the performance of BPGA, it was applied to a test dataset comprising of the complete genome sequences of 28 strains of *Streptococcus pyogenes* species, downloaded from NCBI Genome Database (Supplementary Table S1a). Through initial input preparation process, BPGA generated a file containing 49891 annotated sequences from 28 *S. pyogenes* genomes, which was then used as the input file for clustering.

Though BPGA uses USEARCH as the default clustering tool, here we have used all three options for clustering, namely USEARCH, CD-HIT and OrthoMCL, in parallel, to test the compatibility of BPGA with these three clustering tools.

All three clustering outputs were processed independently to perform pan-genome analysis on the same dataset. Using 50% sequence identity as the cut-off value, USEARCH, CD-HIT and OrthoMCL yielded 2790, 2743 and 2762 distinct clusters respectively, out of which 913, 914 and 855 clusters respectively contain at least one gene from each genome and thus construct the core genome of *S. pyogenes*.

It should be noted that pan genome analysis of *S. pyogenes* genomes was conducted earlier by two different groups. Lefébure and Stanhope carried out the pan genome analysis of the genus *Streptococcus* using a dataset of 26 *Streptococcus* genomes, 11 of which belonged to *S. pyogenes*^8^. The other study was conducted by Zhao *et al.*, where 11 *S. pyogenes* strains were employed to evaluate the performance of the pan genome analysis pipeline PGAP^8^,^28^. With a view to compare the performance of BPGA, we have used this dataset of 11 *S. pyogenes* genomes as a control dataset. Both the pan genome and core genome sizes determined by BPGA (2530 & 1359 respectively) (Supplementary Table S1b) are comparable to those reported by these two previous studies (pan genome sizes 2448/2710 & core genome sizes 1376/1370) and in all three cases, *S. pyogenes* pan-genome is open. However, time taken by BPGA to carry out the analysis is much shorter than that taken by the methods employed in these previous reports. Furthermore, many of the downstream analyses conducted by BPGA such as COG distribution, KEGG pathway analyses, MLST phylogeny or exclusive absence of genes in different *S. pyogenes* strains were not carried out earlier in these two reports^8^,^28^.

### Pan-genome profile estimation and statistics

**Figure 2:**
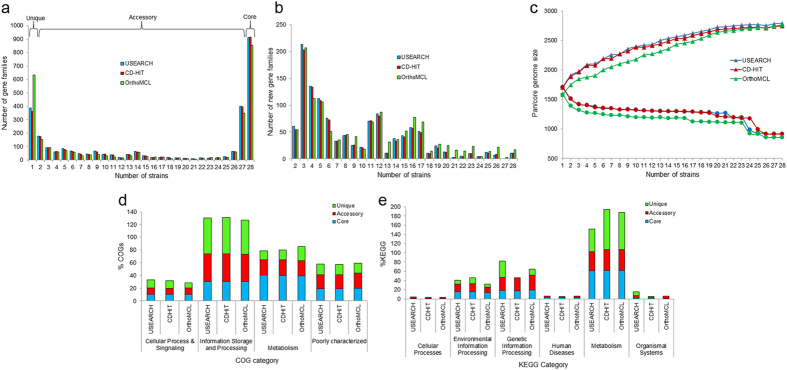
Overview of the results generated by BPGA using 28 strains of *S. pyogenes*. (**a**) The gene family frequency spectrum. (**b**) New gene family distribution after sequential addition of each genome to the analysis. (**c**) The pan genome profile trends obtained using clustering tools- USEARCH, CD-HIT and OrthoMCL. (**d**) COG distribution of core, accessory and unique genes. (**e**) KEGG distribution of core, accessory and unique genes.

Gene family distribution and new gene distribution of *S. Pyogenes* pan genome are shown in Fig. 2a,b respectively. Figure 2c shows the pan genome profile trends obtained using clustering tools- USEARCH, CD-HIT and OrthoMCL; and the pan genome profile curves are shown in the Supplementary Fig. S1. The empirical power law equations and exponential equations were used for extrapolation of the pan and core genome curves respectively, as displayed in the respective plots generated by three clustering tools (Supplementary Fig. S1)^28^. In all three cases, the pan-genome curve has almost reached a plateau, indicating that the *Streptococcus pyogenes* pan-genome would probably close in near future. As can be seen from these figures, the outputs of USEARCH and CD-HIT are quite similar to one another, but those of OrthoMCL differ slightly. This could be due to the fact that OrthoMCL estimates the unique gene clusters separately while USEARCH and CD-HIT clusters all the genes at a time. Genome wise statistics (i.e., distribution of core, accessory, unique and exclusively absent genes) have also been generated by BPGA individually for three clustering tools (Supplementary Tables S2–S4).

### Exclusive presence and absence of genes

As mentioned above, the *Exclusive Gene Family Analysis* is a unique feature of BPGA, which enables extraction of sequences of not only the genes that are exclusively present (unique or singletons), but also the gene families that are exclusively absent in a particular genome of the dataset (i.e., genes present in all but one of the genomes under study). The number of genes exclusively present (unique genes or singletons) and exclusively absent in each strain of *S. pyogenes* dataset are shown in last two columns of Supplementary Tables S2–S4. Intriguingly enough, the number of exclusively absent genes appeared to be exceptionally high in *S. pyogenes STAB901* and *S. pyogenes M1 476* with all three clustering tools. Both these genomes are significantly shorter in length as compared to other *S. pyogenes* under study (Supplementary Table S1a) and the number of total genes and accessory genes are also relatively less in these two genomes (Supplementary Tables S2–S4). Though to our knowledge, there has been no direct evidence for genome reduction in *S. pyogenes*, considering the fact that host restricted commensal and pathogenic bacteria often undergo genome reduction and maintain relatively small genome sizes as compared to the free-living bacterial species, it would be intriguing to examine whether these two genomes are undergoing the process of genome reduction^38^,^39^,^40^. It may be noted here that the *S. pyogenes strain 7F7* also has relatively less number of annotated genes, but all three clustering tools have yielded only one exclusively absent gene in this strain.

### Sequence extraction

Protein sequences for representatives of core (913), accessory (1490) and unique (387) orthologous clusters were extracted using the *Pan Genome Sequence Extraction* module of the BPGA pipeline, as FASTA files. In addition, genome wise sequences for all proteins of the core, accessory, unique and exclusively absent clusters have also been extracted to respective files. Sequences for 928 genes having atypical GC contents have also been extracted.

### Phylogeny based on MLST, core and pan-genome

**Figure 3:**
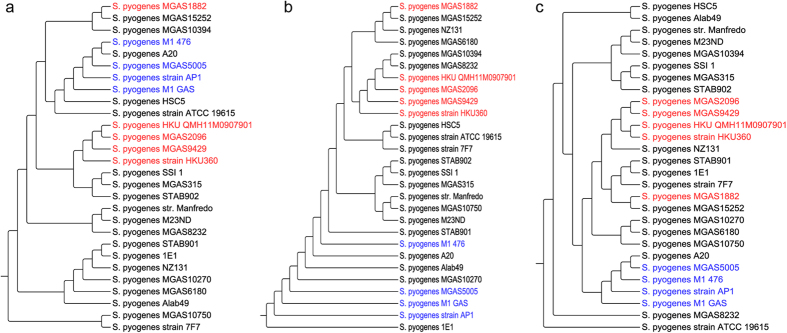
Phylogenetic analysis by BPGA using 28 strains of *S. pyogenes* based on. (**a**) concatenated core genes (**b**) concatenated housekeeping genes (MLST) (**c**) binary pan-matrix. (Blue: Group M1 strains and Red: Group M12 strains).

BPGA generated three types of phylogenetic trees: one, based on concatenated core gene alignments, another based on *in silico* MLST of housekeeping genes and the last one using pan-matrix (i.e., binary gene presence/absence (1/0) matrix). These three types of trees of *S. pyogenes* strains generated using USEARCH clustering are shown in Fig. 3. As expected, there are some variations across the trees generated by different types of phylogenetic approaches. Pan and core genome based trees using other two clustering tools are shown in Supplementary Fig. S2.

### Function and pathway mapping of genes

The *Pan Genome Functional Analysis* module of BPGA has been used for COG function and KEGG pathway mapping of representative protein sequences of core, accessory and unique clusters of the *S. pyogenes* strains under study. Figure 2d shows the COG distribution profiles of the core, accessory and unique gene families. The COG distribution pattern showed involvement of more core genes in *carbohydrate transport and metabolism* and *translation, ribosomal structure and biogenesis*, while accessory and unique genes appear to be enriched in *Transcription, Replication, recombination and repair* and *Cell wall/membrane/envelope biogenesis* related functions. Out of 2790 gene clusters, BPGA could map 1218 (43.7%) to KEGG pathways. As expected, KEGG assignments from BPGA revealed overall higher representation of *metabolism* related pathways (Fig. 2e). BPGA also provides detailed COG and KEGG distribution for *S. pyogenes* clusters generated by USEARCH, CD-HIT and OrthoMCL (Supplementary Figs S3, S4).

### Atypical GC-content Analysis

This module identifies the genes, the G + C-content of which deviate from the average G + C-content of the respective genome by more than 2σ (or 3σ, as defined by the user), where σ is the standard deviation from the average G + C-content^35^,^36^. In a microbial genome, the G + C-content of the native genes (i.e., the genes vertically inherited from its parent organism), in general, does not vary appreciably, the genes with atypical G + C-content are likely to be acquired through horizontal gene transfer^35^,^36^. The last column of Supplementary Table S1a shows the number of such atypical genes found in each *S. pyogenes* genome. These potential alien sequences are then categorized as the core, accessory and unique genes.

### Subset Analysis

We have carried out the pan genome profile analysis for (i) four strains belonging of M-1 serotype and (ii) four strains of M-12 serotypes of *S. pyogenes* test dataset. The results obtained for gene family distribution in these two subsets is shown in Supplementary Fig. S5, while the gene-families present exclusively in M-1 and M-12 serotypes are enlisted in Supplementary file 2^41^,^42^,^43^,^44^. This analysis clearly illustrates how BPGA enables identification of serotype-specific gene families. Needless to say, similar type of analysis applied to the sub-groups of organisms with distinct ecological or pathogenic traits may help to identify niche-specific or virulence-specific genes in bacteria.

### Performance and feature comparisons with other tools

Exact comparison of performances of various pan-genome analysis tools with BPGA is not possible because they are not identical in their tasks and features. But there are considerable advantages like ease of use and improved performance (Table 2). We have analyzed the performance and memory requirements of BPGA for various datasets (Supplementary Table S5). Smaller datasets (24 and 28 strains; not tiny though) took only 3 to 4 minutes for all analyses together. We also tested the performance for extremely large datasets (1000 strains, and 1000 random bacterial genomes); none of them exceeded 420 minutes and 3.5 gigabytes memory (RAM).

## Discussion

With rapid advances in NextGen sequencing techniques and metagenomics, bacterial genomics has transitioned from single-genome studies to cross-genome comparison of hundreds to thousands organisms at different scales of phylogenetic resolution. As a consequence, pan genome studies have widened their scope from species to higher taxonomic levels, necessitating development of faster analysis tools capable of processing larger datasets. In an attempt to speed up the whole process of analysis, the fastest tool for gene family clustering, USEARCH, has been incorporated as the default tool in BPGA pipeline. This has decreased the redundancy of genes in the dataset by grouping the genes into orthologous clusters and using only the representatives for comparative studies. As illustrated in the present report, BPGA outperforms most of the existing pan genomic tools in the speed of operation (Table 2).

Another major issue in pan genome studies is purposeful use of the binary pan matrix data for downstream functional analyses. In addition to all types of analyses offered by currently available tools, BPGA contains certain novel features like Exclusive Gene Family Analysis, Atypical GC Content Analysis and Subset Analysis etc. The option for exclusive presence and absence of genes in one or more genomes in the dataset paves the way to probe into the genetic basis of various microbial phenotypes, such as emergence of virulence or symbiosis, adaptation to specific niches and so on. Exclusive absence of genes in one or more particular strains may also hint towards reductive evolution of the respective genomes– an event often observed in obligatory host-associated microbes^38^,^39^. The module on subset analysis, when applied to the sub-groups of organisms with distinct serological, ecological or pathogenic traits may also provide clues for bacterial genome dynamics and evolution under specific conditions.

Identification of genes with atypical GC contents point towards a probable events of horizontal acquisition of genes from other bacteria in course of evolution or as an adaptation strategy^35^,^36^. To our knowledge, BPGA is the only pan genomic tool that can identify such genes or gene clusters. It may be noted that the atypical GC-content alone cannot confirm the alien origin of any bacterial gene. There are some other parameters like codon usage, oligonucleotide usage etc. which should be checked before coming to any conclusion regarding the possibility of horizontal acquisition of these genes. Nevertheless, the *Atypical GC Content Analysis* module may give some clues on the potential cases of Horizontal Gene Transfer in a microbial genome^35^,^36^.

BPGA has overcome the shortcomings in earlier pan genome analysis tools. It provides user defined input options unlike online resource (e.g. PanOCT) with limited strains and/or lack of newly sequenced strains. It even works with most recent bacterial draft genomes from The Human Microbiome Project, submitted to GenBank. As mentioned above, BPGA provides important downstream analysis options which Panseq, PanGP and Roary lacked. BPGA has significantly higher speed of analysis as compared to that of PGAP or ITEP. Integration of support for diverse clustering methods (USEARCH, CD-HIT and OrthoMCL) has increased the scope of BPGA. Other notable features of BPGA includes minimum running prerequisites, ease of handling, user friendly command line interface, freedom for user to select method for clustering, high quality image output and efficiency in terms of time cost.

### Software availability

BPGA is freely available at: http://sourceforge.net/projects/bpgatool/ and http://www.iicb.res.in/bpga/index.html.

## Additional Information

**How to cite this article**: Chaudhari, N. M. *et al.* BPGA- an ultra-fast pan-genome analysis pipeline. *Sci. Rep.* **6**, 24373; doi: 10.1038/srep24373 (2016).

## Supplementary Material

Supplementary Information

## Figures and Tables

**Table 1:** Description of BPGA Pipeline.

Features	Description	Tools/scripts	Notes	Equivalent tools.	Citation
Preparation step	Preprocessing of raw files (.faa, .fsa or any fasta or .gbk) leading to a single *input file* required for clustering.	BPGA script	BPGA modifies the files by inserting genome ID into the sequence headers.	NA	This study
Clustering	It is used to cluster genes based on sequence similarity into orthlogous clusters.	USEARCH#, CD-HIT*, OrthoMCL*.	USEARCH is fastest clustering tool so far. BPGA uses it as default clustering tool and can also process the clusters from other two.	Roary, PGAP, PGAT, ITEP, Panseq.	[25,^27^, ^28^, ^29^,^45^]
Matrix Generation (Pan-Matrix)	It generates 1,0–binary presence/ absence matrix from orthlogous clusters.	BPGA script	BPGA script checks the presence or absence of genes from the individual strains and writes in the form of matrix.	Roary, PanGP, PGAP.	[26, 27, 28]
Pan-Genome Profile Analysis	Calculates shared genes after stepwise addition of each individual genome. This trend can be plotted as Core or Pan-genome Profile Curves.	BPGA script, gnuplot.	BPGA script calculates such trends taking different permutations/combinations of genomes.	Roary, PanGP, PGAP.	[26, 27, 28]
Phylogeny Construction	*Pan Phylogeny*: Generates a phylogenetic tree based on pan-matrix data. *Core/MLST Phylogeny*: Generates a phylogenetic tree based on concatenated core/housekeeping gene alignments.	BPGA script, MUSCLE#, Librsvg.	BPGA script concatenates the core sequences from all strains and converts pan-matrix into Newick tree. MUSCLE is faster and more accurate alignment and tree generator tool.	Roary, PGAP, Panseq, ITEP.	[25,^27^, ^28^, ^29^]
Function and Pathway† Analysis	COG and KEGG Assignments on the basis of best hits with respective reference databases.	USEARCH#, BPGA script, gnuplot.	Best hits are processed to get the % occurrences for all COG & KEGG pathway categories.	*COG*: PGAP, PGAT,ITEP. *KEGG Analysis*: None	[28,^29^,^45^]
Pan-Genome Statistics†	It provides genome wise core, accessory, unique and exclusively absent gene counts.	BPGA script	Gives an idea about contribution of each strain to the pan-genome.	None	This study
Atypical GC Content Analysis†	Identifies genes with substantial high or low GC content from their genomic GC content.	BPGA script	Applicable only if Genbank files are used as input.	None	This study
Subset Analysis†	Divides the original dataset into user defined smaller subsets and performs default pan genomic analyses.	BPGA script	The subsets may be based on pathogenic potential, habitat, taxonomical groups or any other criteria.	None	This study
Exclusive gene absence†	Identifies the clusters showing exclusive absence of a gene from the specific strain.	BPGA script	Sequences of such clusters are given in output file.	None	This study

^#^Automated by BPGA script.

^*^Supported outputs.

^†^These are novel features by BPGA, NA-Not Applicable.

**Table 2:** Comparison of BPGA with other tools currently available for pan-genome analysis.

Features	BPGA	Roary^27^	PanGP^26^	PGAP^28^	PGAT^45^	Panseq^25^	ITEP^29^
Input file/s	.GBK/.FAA/Matrix	.gff	Cluster/ Matrix	.FAA, .FFN & .PTT	×	Contig file	×
**Exclusively absent genes**	✓	×	×	×	×	×	×
**Subset Analysis**	✓	×	×	×	×	×	×
**Atypical GC analysis**	✓	×	×	×	×	×	×
**Distribution of core, accessory and unique genome**	✓	×	×	×	×	×	×
**KEGG pathway distribution**	✓	×	×	×	×	×	×
Pan-genome profile analysis	✓	✓	✓	✓	×	×	×
Size of core and pan-genome	✓	✓	×	×	×	✓	×
Extraction of core, accessory and unique genome sequence	✓	✓	×	×	×	×	✓
Evolutionary analysis	✓	✓	×	✓	×	✓	✓
Functional distribution analysis (COG)	✓	×	×	✓	✓	×	✓
Protein/gene clustering	✓	✓	×	✓	✓	✓	✓
Input data from user	✓	✓	✓	✓	×	✓	×
Operating System	Windows, Linux and any Perl supported OS.	Linux	Windows & Linux	Linux	NA	Windows & Linux	Linux
Mode of program	Standalone	Standalone	Standalone	Standalone	Online Database	Online & Standalone	Standalone
Test dataset	*S. pyogenes*	*S. typhi*	*E. coli*	*S. pyogenes*	NA	*S. pyogenes*	*Enterobacteria*
No. of genomes in test dataset	28	24	30	11	NA	11	24
Time cost	~3 m	>6 m	48 sec	29–200 m	NA	~10 m	>1 hr
Performance	Very Fast	Fast	Fast	Slow	Slow	Slow	Slow

Note: Features in bold are unique to BPGA.
