# Supplementary material for: BPGA- an ultra-fast pan-genome analysis pipeline
Source: Sci Rep. 2016 Apr 13;6:24373. doi: 10.1038/srep24373 (PMC4829868; doi:10.1038/srep24373)
Supplement: Supplementary Information [file srep24373-s1.pdf]

BPGA - an ultra-fast pan-genome analysis pipeline

Narendrakumar M. Chaudhari[1,+], Vinod Kumar Gupta[1,+] and Chitra Dutta[1,*]

[1]Structural Biology & Bioinformatics Division, CSIR- Indian Institute of Chemical Biology, 4, Raja S. C. Mullick Road, Kolkata 700032, India.

[*]Corresponding Author, C.D. (cdutta@iicb.res.in).

[+]These authors contributed equally to this work.

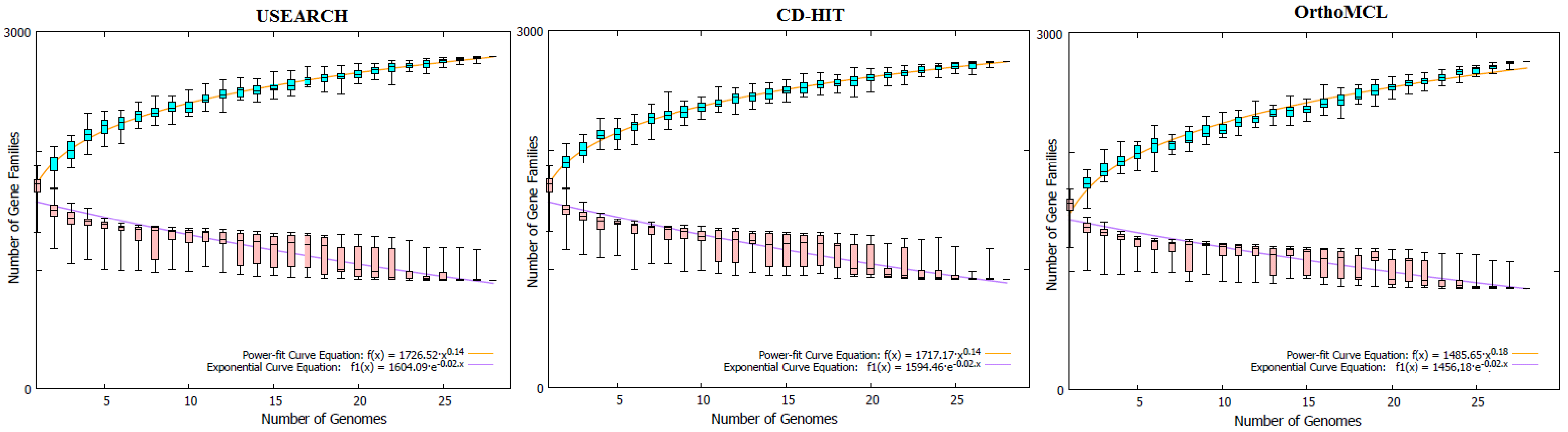

Supplementary Figure S1: Pan-genome profile curves for 28 strains of *Streptococcus pyogenes*.

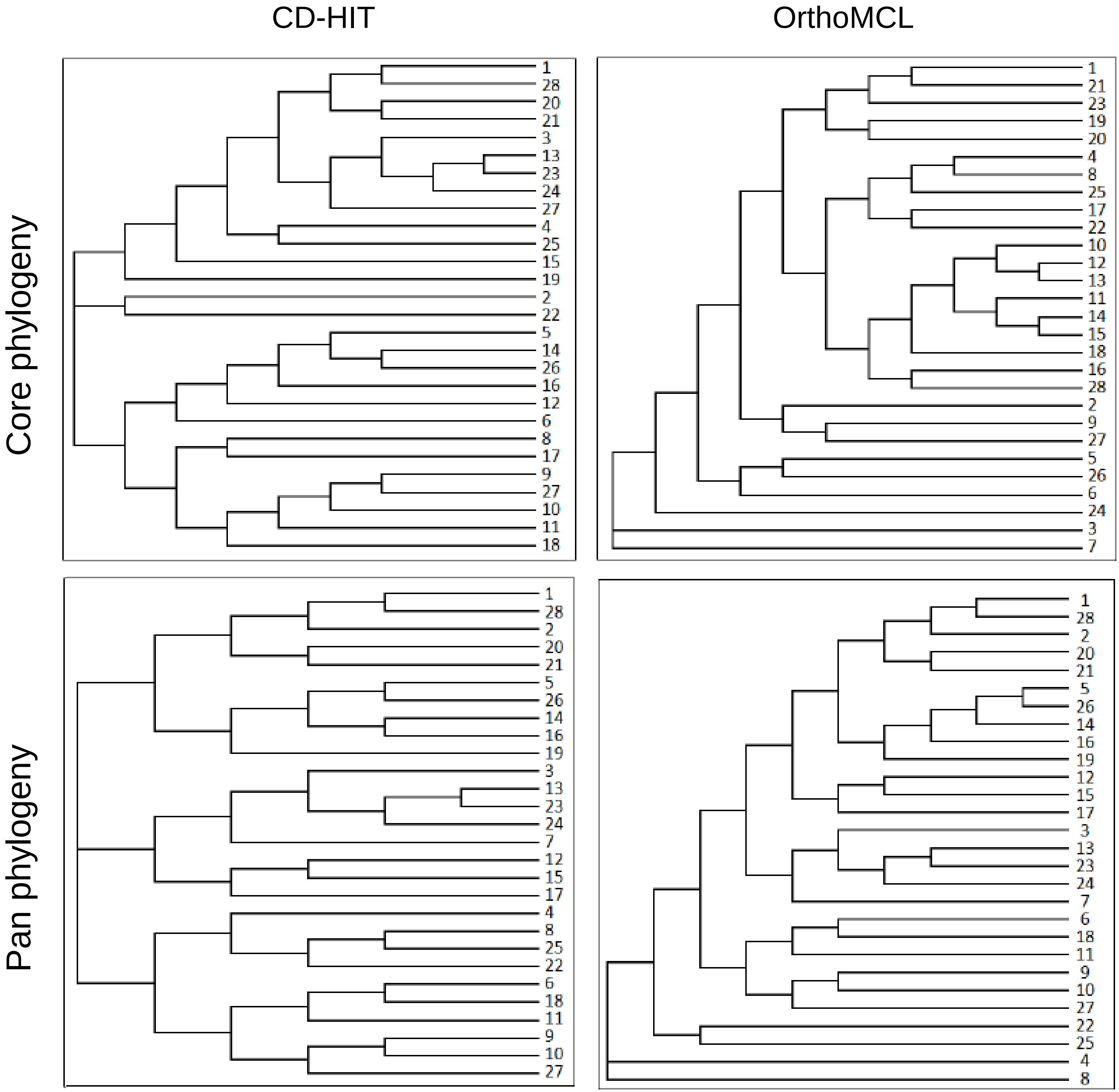

Supplementary Figure S2: Phylogenetic trees based on core & pan genome for 28 strains of *Streptococcus pyogenes*.

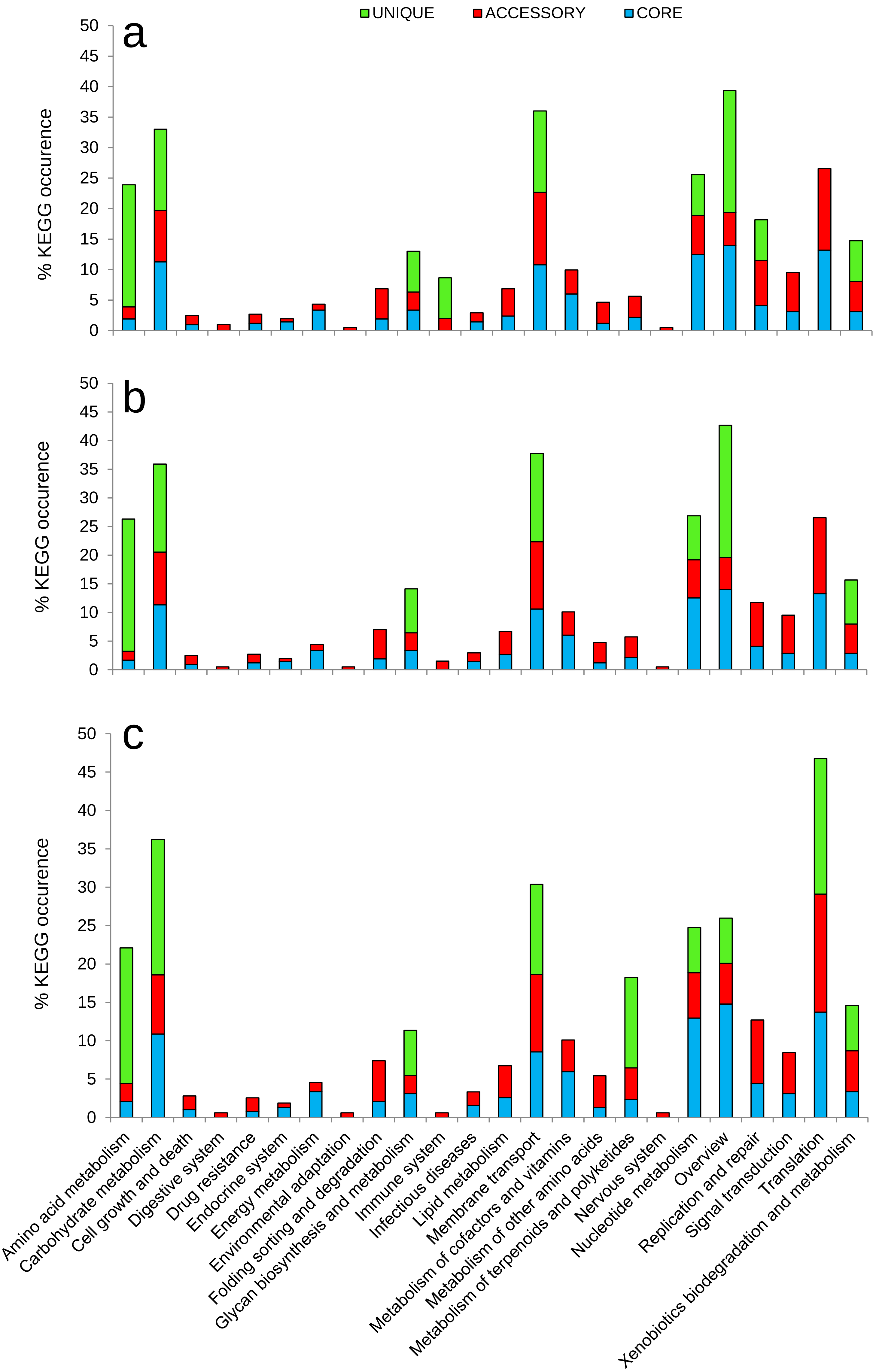

Supplementary Figure S3: KEGG pathway distribution of *S. pyogenes* pan genome from (a) USEARCH, (b) CD-HIT and (c) OrthoMCL.

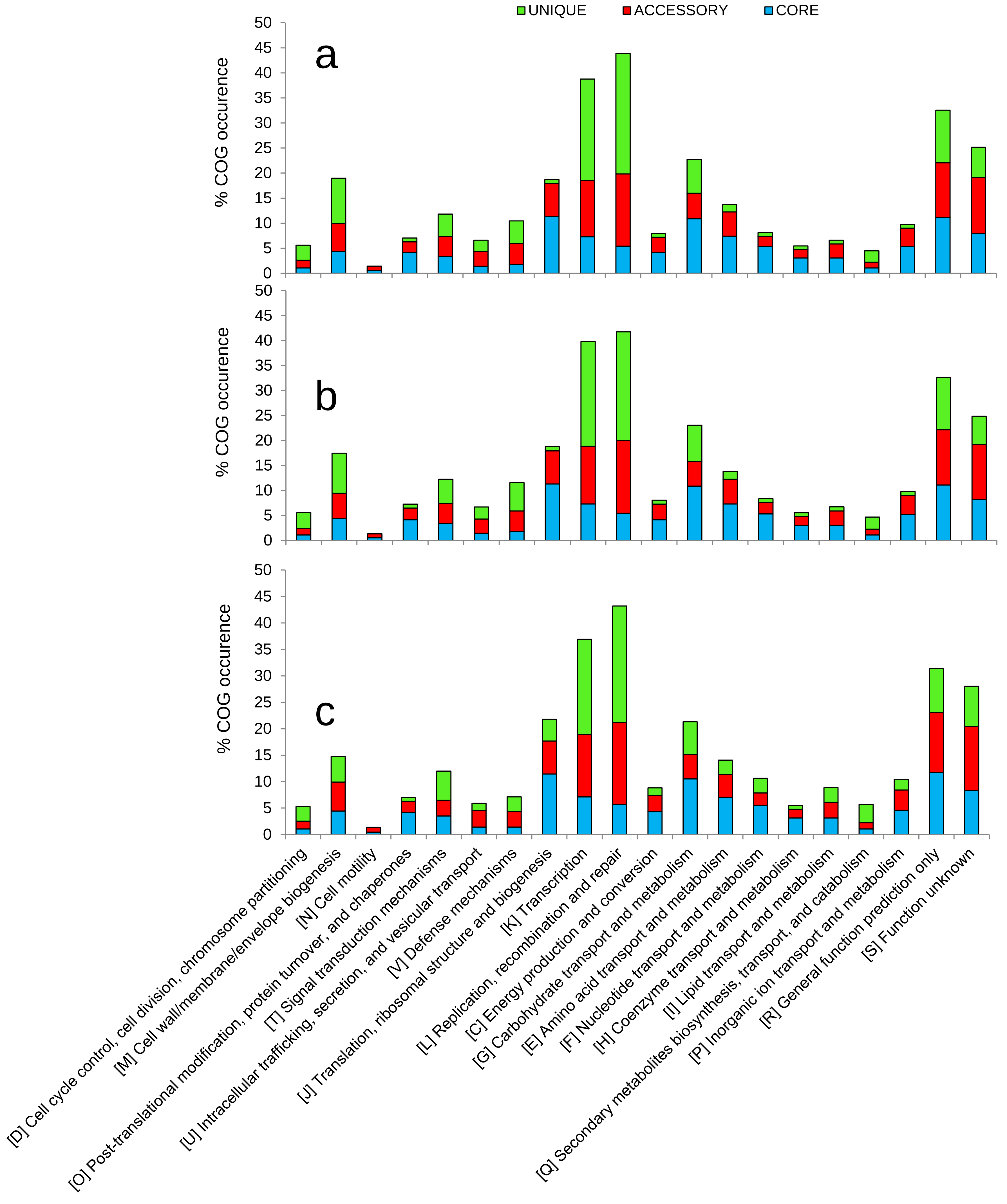

Supplementary Figure S4: COG distribution of *S. pyogenes* pan genome from (a) USEARCH, (b) CD-HIT and (c) OrthoMCL.

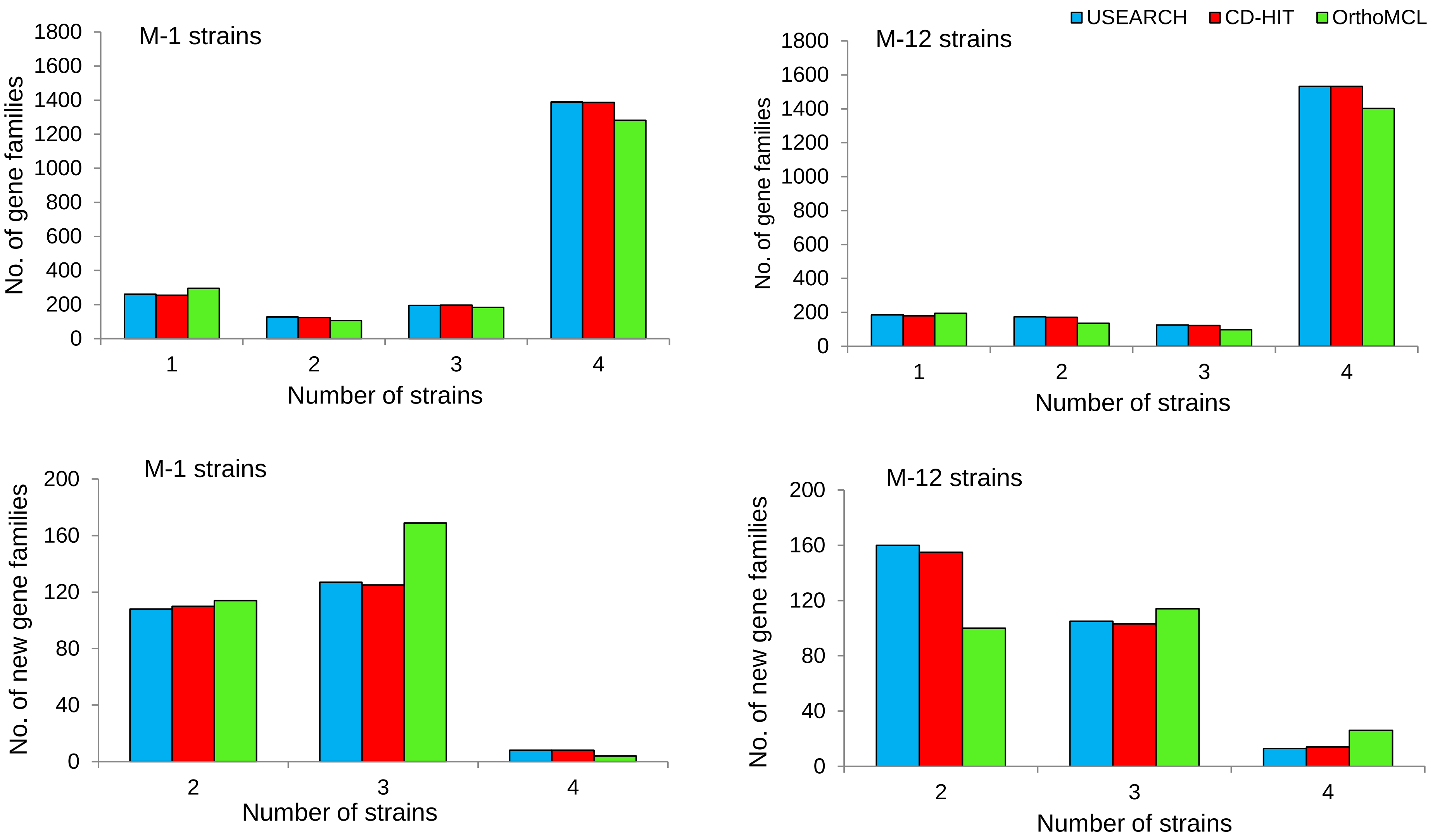

Supplementary Figure S5: Comparison of gene family distribution among strains (upper panel) and addition of new genes into pan-genome (lower panel); between two groups: M-1 strains and M-12 strains of *Streptococcus pyogenes* species.

Table S1a: Test dataset.

Sr. No.	Organism name	No. of proteins	Genomic G+C (%)	No. of Atypical Genes
1	*Streptococcus pyogenes 1E1*	1652	38.6	85
2	*Streptococcus pyogenes strain 7F7*	1550	38.8	92
3	*Streptococcus pyogenes strain AP1*	1836	38.6	122
4	*Streptococcus pyogenes strain ATCC 19615*	1775	38.5	171
5	*Streptococcus pyogenes strain HKU360*	1846	38.4	72
6	*Streptococcus pyogenes M23ND*	1842	38.6	95
7	*Streptococcus pyogenes M1 GAS*	1696	38.6	130
8	*Streptococcus pyogenes MGAS8232*	1839	38.5	121
9	*Streptococcus pyogenes MGAS315*	1865	38.6	187
10	*Streptococcus pyogenes SSI-1*	1859	38.5	150
11	*Streptococcus pyogenes MGAS10394*	1886	38.6	168
12	*Streptococcus pyogenes MGAS6180*	1894	38.3	128
13	*Streptococcus pyogenes MGAS5005*	1865	38.5	165
14	*Streptococcus pyogenes MGAS9429*	1877	38.3	102
15	*Streptococcus pyogenes MGAS10270*	1987	38.3	92
16	*Streptococcus pyogenes MGAS2096*	1898	38.6	121
17	*Streptococcus pyogenes MGAS10750*	1978	38.3	128
18	*Streptococcus pyogenes str. Manfredo*	1745	38.7	134
19	*Streptococcus pyogenes NZ131*	1700	38.6	134
20	*Streptococcus pyogenes MGAS15252*	1662	38.6	69
21	*Streptococcus pyogenes MGAS1882*	1691	38.6	72
22	*Streptococcus pyogenes Alab49*	1773	38.6	148
23	*Streptococcus pyogenes A20*	1828	38.5	169
24	*Streptococcus pyogenes M1 476*	1571	38.7	141
25	*Streptococcus pyogenes HSC5*	1744	38.5	183
26	*Streptococcus pyogenes HKU QMH11M0907901*	1865	38.4	122
27	*Streptococcus pyogenes STAB902*	1809	38.6	124
28	*Streptococcus pyogenes STAB901*	1358	38.7	73

M-1 Strains, **M-12 Strains**.

Table S1b: Additional Test Dataset **-** Distribution of core, accessory, unique and exclusively absent genes among 11 *S. pyogenes* strains

Sr. No.	Organism name	No. of core genes	No. of accessory genes	No. of unique genes	No. of exclusively absent genes
1	*Streptococcus pyogenes M1 GAS*	1359	274	7	27
2	*Streptococcus pyogenes MGAS8232*	1359	353	23	8
3	*Streptococcus pyogenes MGAS315*	1359	384	6	4
4	*Streptococcus pyogenes SSI 1*	1359	379	18	6
5	*Streptococcus pyogenes MGAS10394*	1359	361	65	10
6	*Streptococcus pyogenes MGAS6180*	1359	446	19	0
7	*Streptococcus pyogenes MGAS5005*	1359	405	7	3
8	*Streptococcus pyogenes MGAS9429*	1359	400	30	1
9	*Streptococcus pyogenes MGAS10270*	1359	469	42	1
10	*Streptococcus pyogenes MGAS2096*	1359	339	59	7
11	*Streptococcus pyogenes MGAS10750*	1359	428	72	3

Table S2: Distribution of core, accessory, unique and exclusively absent genes among all 28 *S. pyogenes* strains using USEARCH clustering tool.

Genome id	Organism name	No. of core genes	No. of accessory genes	No. of unique genes	No. of exclusively absent genes
1	*Streptococcus pyogenes 1E1*	913	697	5	2
2	*Streptococcus pyogenes strain 7F7*	913	601	2	1
3	*Streptococcus pyogenes strain AP1*	913	804	12	3
4	*Streptococcus pyogenes strain ATCC 19615*	913	765	8	1
5	*Streptococcus pyogenes strain HKU360*	913	821	5	2
6	*Streptococcus pyogenes M23ND*	913	779	21	2
7	*Streptococcus pyogenes M1 GAS*	913	722	1	13
8	*Streptococcus pyogenes MGAS8232*	913	805	13	2
9	*Streptococcus pyogenes MGAS315*	913	834	1	0
10	*Streptococcus pyogenes SSI 1*	913	828	13	3
11	*Streptococcus pyogenes MGAS10394*	913	814	55	6
12	*Streptococcus pyogenes MGAS6180*	913	894	14	0
13	*Streptococcus pyogenes MGAS5005*	913	850	2	0
14	*Streptococcus pyogenes MGAS9429*	913	853	22	0
15	*Streptococcus pyogenes MGAS10270*	913	922	35	0
16	*Streptococcus pyogenes MGAS2096*	913	788	56	3
17	*Streptococcus pyogenes MGAS10750*	913	900	45	1
18	*Streptococcus pyogenes str. Manfredo*	913	751	8	10
19	*Streptococcus pyogenes NZ131*	913	721	21	4
20	*Streptococcus pyogenes MGAS15252*	913	701	1	1
21	*Streptococcus pyogenes MGAS1882*	913	739	3	1
22	*Streptococcus pyogenes Alab49*	913	775	5	4
23	*Streptococcus pyogenes A20*	913	841	8	0
24	*Streptococcus pyogenes M1 476*	913	609	4	65
25	*Streptococcus pyogenes HSC5*	913	729	10	16
26	*Streptococcus pyogenes HKU QMH11M0907901*	913	836	7	0
27	*Streptococcus pyogenes STAB902*	913	797	0	2
28	*Streptococcus pyogenes STAB901*	913	391	11	257

Table S3: Distribution of core, accessory, unique and exclusively absent genes among all 28 *S. pyogenes* strains using CD-HIT clustering tool.

Genome id	Organism name	No. of core genes	No. of accessory genes	No. of unique genes	No. of exclusively absent genes
1	*Streptococcus pyogenes 1E1*	914	696	3	2
2	*Streptococcus pyogenes strain 7F7*	914	596	3	1
3	*Streptococcus pyogenes strain AP1*	914	792	13	3
4	*Streptococcus pyogenes strain ATCC 19615*	914	759	8	1
5	*Streptococcus pyogenes strain HKU360*	914	813	4	2
6	*Streptococcus pyogenes M23ND*	914	773	19	2
7	*Streptococcus pyogenes M1 GAS*	914	717	1	13
8	*Streptococcus pyogenes MGAS8232*	914	797	12	2
9	*Streptococcus pyogenes MGAS315*	914	825	1	0
10	*Streptococcus pyogenes SSI 1*	914	817	11	3
11	*Streptococcus pyogenes MGAS10394*	914	807	54	6
12	*Streptococcus pyogenes MGAS6180*	914	894	11	0
13	*Streptococcus pyogenes MGAS5005*	914	845	2	0
14	*Streptococcus pyogenes MGAS9429*	914	849	18	0
15	*Streptococcus pyogenes MGAS10270*	914	916	32	0
16	*Streptococcus pyogenes MGAS2096*	914	781	56	3
17	*Streptococcus pyogenes MGAS10750*	914	890	43	1
18	*Streptococcus pyogenes str. Manfredo*	914	742	7	10
19	*Streptococcus pyogenes NZ131*	914	723	17	4
20	*Streptococcus pyogenes MGAS15252*	914	696	1	2
21	*Streptococcus pyogenes MGAS1882*	914	733	3	1
22	*Streptococcus pyogenes Alab49*	914	767	4	4
23	*Streptococcus pyogenes A20*	914	834	8	0
24	*Streptococcus pyogenes M1 476*	914	608	4	64
25	*Streptococcus pyogenes HSC5*	914	723	9	16
26	*Streptococcus pyogenes HKU QMH11M0907901*	914	828	8	0
27	*Streptococcus pyogenes STAB902*	914	790	0	2
28	*Streptococcus pyogenes STAB901*	914	390	11	254

Table S4: Distribution of core, accessory, unique and exclusively absent genes among all 28 *S. pyogenes* strains using OrthoMCL clustering tool.

Genome id	Organism name	No. of core genes	No. of accessory genes	No. of unique genes	No. of exclusively absent genes
1	*Streptococcus pyogenes 1E1*	854	626	9	2
2	*Streptococcus pyogenes strain 7F7*	854	538	5	1
3	*Streptococcus pyogenes strain AP1*	854	726	22	3
4	*Streptococcus pyogenes strain ATCC 19615*	854	672	17	2
5	*Streptococcus pyogenes strain HKU360*	854	722	13	2
6	*Streptococcus pyogenes M23ND*	854	637	36	3
7	*Streptococcus pyogenes M1 GAS*	854	649	5	8
8	*Streptococcus pyogenes MGAS8232*	854	698	22	2
9	*Streptococcus pyogenes MGAS315*	854	746	10	0
10	*Streptococcus pyogenes SSI 1*	854	730	14	3
11	*Streptococcus pyogenes MGAS10394*	854	697	59	5
12	*Streptococcus pyogenes MGAS6180*	854	766	30	0
13	*Streptococcus pyogenes MGAS5005*	854	728	24	0
14	*Streptococcus pyogenes MGAS9429*	854	715	23	0
15	*Streptococcus pyogenes MGAS10270*	854	787	47	0
16	*Streptococcus pyogenes MGAS2096*	854	664	75	4
17	*Streptococcus pyogenes MGAS10750*	854	762	62	1
18	*Streptococcus pyogenes str. Manfredo*	854	662	12	8
19	*Streptococcus pyogenes NZ131*	854	639	24	4
20	*Streptococcus pyogenes MGAS15252*	854	608	11	2
21	*Streptococcus pyogenes MGAS1882*	854	641	16	1
22	*Streptococcus pyogenes Alab49*	854	643	15	4
23	*Streptococcus pyogenes A20*	854	696	23	0
24	*Streptococcus pyogenes M1 476*	854	550	4	53
25	*Streptococcus pyogenes HSC5*	854	631	13	14
26	*Streptococcus pyogenes HKU QMH11M0907901*	854	716	23	0
27	*Streptococcus pyogenes STAB902*	854	725	3	2
28	*Streptococcus pyogenes STAB901*	854	328	17	223

Table S5: Performance of BPGA on different datasets.

No. of Genomes	Total no. of proteins	Number of protein clusters	Time cost	Maximum RAM used [#] (Mb)
24[$]	109,478	9,431	3m50s	450
28	49,891	2,790	2m30s	310
200	332,718	156,328	17m[@]	1100
500	979,238	409,451	122m[@]	2000
1000	1,769,242	680,832	420m[@]	3000
1000[$]	4,405,532	22,496	111m[@]	3400

[$]Strains of *Salmonella enterica* species,[@] Time excluding KEGG/COG function analysis, [#]Intel CORE i5 processor with 4 Gb RAM installed. USEARCH is used in all the cases as default clustering tool.
